# A novel approach: a pancreatic duct stent removable with the nasobiliary catheter

**DOI:** 10.1055/a-2760-9468

**Published:** 2026-01-08

**Authors:** Haiyong Long, Ping Wang, Wenguang Yang, Yuhong Ren, Bin Yang, Sichao Wen, Mingwen Guo

**Affiliations:** 1 Department of Gastroenterology, Qionglai Medical Center Hospital, Qionglai, China

Post-endoscopic retrograde cholangiopancreatography (ERCP) pancreatitis (PEP) is one of the most common complications of ERCP. Prophylactic pancreatic duct (PD) stent placement is an effective measure to reduce its incidence 1 . However, conventional PD stents require follow-up X-rays for the confirmation of spontaneous dislodgement or a second endoscopy for removal, which imposes additional patient discomfort and financial burden 2 . We designed a novel method that allows for the simultaneous removal of a PD stent along with a nasobiliary catheter (NBC) at the bedside, based on clinical needs.

**Fig. 1:**
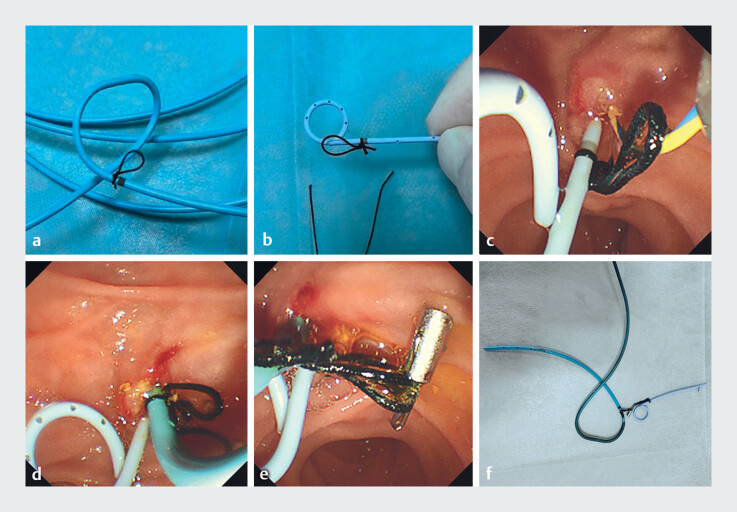
An innovative method: a pancreatic duct stent for removal alongside the nasobiliary catheter. **a** The surgical suture tied at the marked site of the nasobiliary catheter, and the suture loop diameter is 1.5 cm. **b** The surgical suture tied at the marked site of the pancreatic duct stent, and the suture loop diameter is 1.5 cm. **c** Placement of the pancreatic duct stent with the tied suture. **d** Placement of the nasobiliary catheter with the tied suture. **e** Titanium clips used to connect the nasobiliary catheter and pancreatic duct stent. **f** The nasobiliary catheter and pancreatic duct stent connection device.

A 36-year-old woman presented with abdominal pain. Magnetic resonance cholangiopancreatography confirmed choledocholithiasis, and she was scheduled for ERCP ( Video 1 ). Pre-procedure preparation: The NBC and PD stent were pre-modified by tying a surgical suture at their marked positions, forming a loop of approximately 1.5 cm in diameter ( Fig. 1 **a, b** ). During the ERCP procedure, the guidewire was first advanced into the pancreatic duct and was subsequently retained.

Demonstration of the novel technique for the simultaneous removal of the pancreatic duct stent and nasobiliary catheter after endoscopic retrograde cholangiopancreatography.Video 1

Using the double-guidewire technique, the bile duct was successfully cannulated ( Video 1 ). The modified PD stent was deployed over the pancreatic guidewire ( Fig. 1 **c** ). Following balloon extraction of the common bile duct stones ( Video 1 ), the modified NBC was placed ( Fig. 1 **d** ). A titanium clip was used to securely link the suture loops of the NBC and the PD stent ( Fig. 1 **e** ). At 48 hours post-procedure, the patient recovered well with no complications such as PEP, bleeding, or infection. The connected NBC-PD stent assembly was removed gently and completely at the bedside ( Video 1 , Fig. 1 **f** ).

This connected NBC-PD stent assembly offers dual benefits: it secures the PD stent to prevent premature migration and enables its convenient, simultaneous removal with the NBC. This technique effectively obviates the need for additional imaging studies or a repeat endoscopy, presenting a representing and clinically advantageous approach worthy of broader adoption.

Endoscopy_UCTN_Code_TTT_1AR_2AZ
